# Case report: The role and value of radiotherapy in treatment of inflammatory myofibroblastic tumor

**DOI:** 10.3389/fonc.2024.1395787

**Published:** 2024-11-15

**Authors:** Haiwei Guo, Mingyun Jiang, Juanjuan Cai, Ruiqi Liu, Weiping Yao, Xiaodong Liang, Haibo Zhang

**Affiliations:** ^1^ Otolaryngology & Head and Neck Center, Cancer Center, Department of Head and Neck Surgery, Zhejiang Provincial People’ s Hospital, Affiliated People’s Hospital, Hangzhou Medical College, Hangzhou, Zhejiang, China; ^2^ Zhejiang Key Laboratory of Precision Medicine Research on Head & Neck Cancer, Hangzhou, China; ^3^ Zhejiang Provincial Clinical Research Center for Malignant Tumor, Hangzhou, China; ^4^ Department of Oncology, Taixing People’s Hospital, Taixing, China; ^5^ Cancer Center, Department of Pathology, Zhejiang Provincial People’s Hospital (Affiliated People’s Hospital), Hangzhou Medical College, Hangzhou, Zhejiang, China; ^6^ Cancer Center, Department of Radiation Oncology, Zhejiang Provincial People’s Hospital (Affiliated People’s Hospital), Hangzhou Medical College, Hangzhou, Zhejiang, China

**Keywords:** inflammatory myofibroblastic tumor, radiotherapy, radiation dose, concurrent radiochemotherapy, case report

## Abstract

**Background:**

Inflammatory myofibroblastic tumors (IMTs) are rare soft-tissue neoplasms. Accordingly, there is no standardized therapy for unresectable or advanced IMT. Chemotherapy, radiotherapy, and targeted molecular therapy play an important role in unresectable or advanced IMT.

**Case presentation:**

We present a 54-year-old man with a cough and chest distress case report. The thoracic surgeon performed the right upper pulmonary occupying lesion wedge resection and enlarged lymph node excision biopsy. Pathologic diagnosis revealed that the morphology of “right upper lung mass” was considered as Inflammatory Myofibroblastic Tumor (IMT). Radiotherapy was indicated at a high dose: 5400cGy in 27 fractions of 2Gy over 5 weeks were delivered combined with cisplatin. The patient was given a CT/MRI and hematological index every 3 months and experienced no more adverse events. The patient survives with no tumor recurrence as of the last follow-up. Progression-free survival (PFS) exceeded 5 years.

**Conclusions:**

We have reviewed the literature and summarized and discussed the radiotherapy treatment options and challenges for IMT. We first reported high-dose radiotherapy combined with chemotherapy treatment for unresectable IMT. Concurrent radiochemotherapy may be considered an intensive treatment for local progress, local recurrence, and nonresectable IMT patients.

## Background

Inflammatory myofibroblastic tumor (IMT) is a quite rare type of soft-tissue neoplasm. The 2013 World Health Organization classification defined IMT as a mesenchymal neoplasm of intermediate malignancy (1). IMT usually occurs in children, adolescents, and young adults, and the incidence rate of male and female patients is similar (2). Besides, it usually presents as a mass in soft tissue-composed organs and structures, such as the lung, abdomen, pelvis, retroperitoneum, head and neck, central nervous system, and others (3–5). The clinical presentations are various, such as pain, fever, malaise, swelling, weight loss, etc., depending on the IMT location. Moreover, IMT rarely metastasizes, and distant metastatic IMT may only occur in up to 5% of all IMT patients (6).

Because IMT is locally invasive, complete surgical resection is the first choice. However, unresectable or advanced IMT does not have standard therapy. Chemotherapy, radiotherapy, and targeted molecular therapy play an important role in unresectable or advanced IMT. Radiotherapy is usually ineffective for IMT (7). Chemotherapy is also applied in the IMT treatment with good therapeutic effect (8). Besides, the molecular landscape of IMT is essential as they can be targeted for treatment involving several targets, such as ALK, ROS1, PDGFR β, RET, and NTRK (3, 9).

Here, we present a case of IMT located in the lung and mediastinum. This patient accepted subtotal resection and concurrent chemoradiotherapy. Additionally, we reviewed and discussed cases experiences and the role and value of radiotherapy in treatment of inflammatory myofibroblastic tumor.

## Case presentation

**Figure 1:**
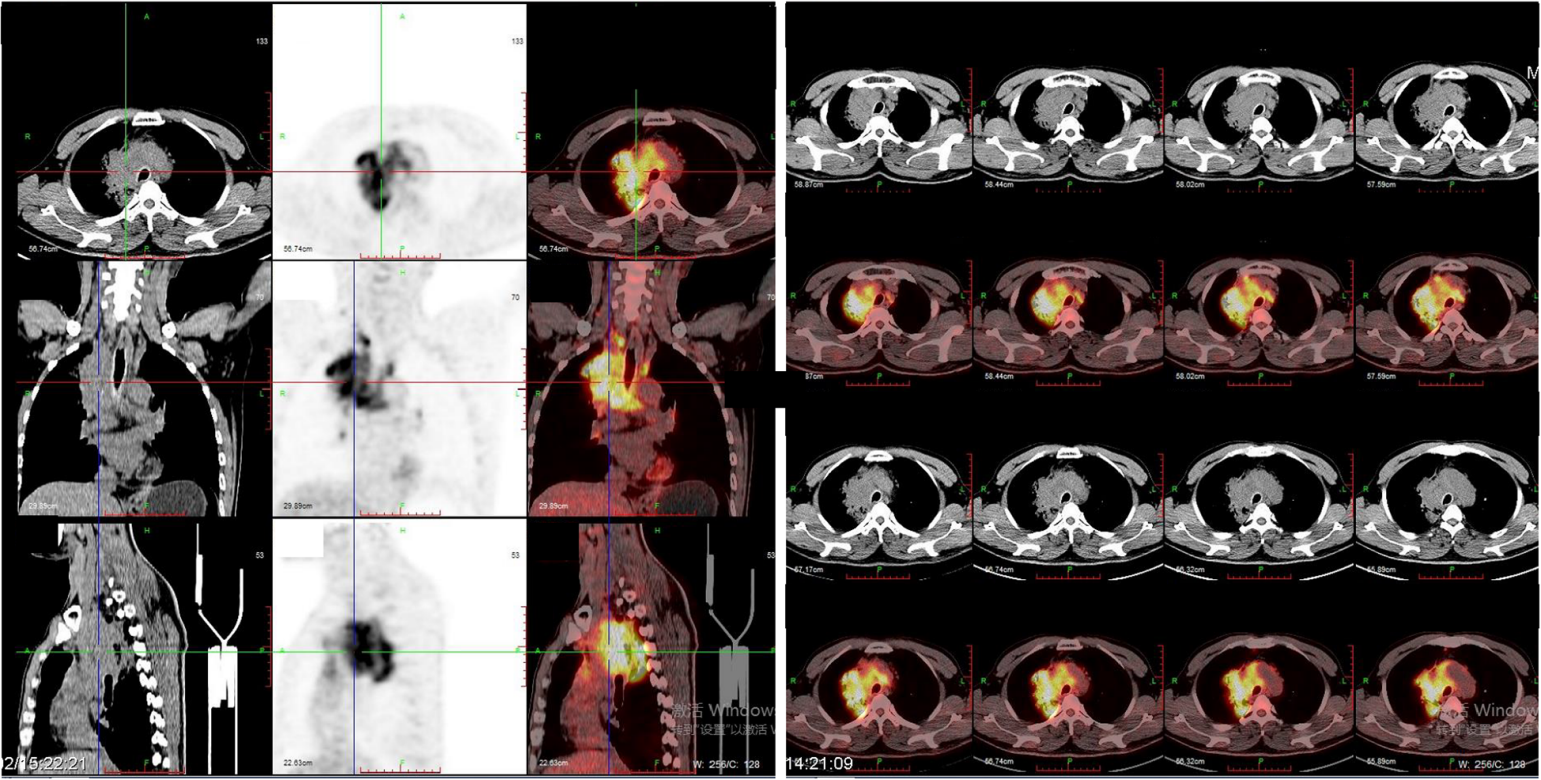
PET\CT images showed a mass (about 7.2 x 6.7 x 6.1 cm) in the superior lobe of right lung with the SUVmax was about 13.4 and lymph node metastasis in hilum of right lung, mediastinum, and right subclavian fossa.

In February 2018, a 54-year-old man presented to the hospital with a cough associated with chest distress. A CT scan was performed and revealed an occupying lesion in the superior lobe of the right lung with enlarged lymph nodes in the mediastinum. The positron emission tomography/computed tomography (PET/CT) showed that the occupying lesion was about 7.2 x 6.7 x 6.1 cm, with the SUVmax about 13.4 and lymph node metastasis in the hilum of the right lung, mediastinum, and right subclavian fossa (**Figure 1**).

In order to identify the pathological type, endobronchial ultrasound (EBUS) and transbronchial needle aspiration (TBNA) were used to assess the lesion organization twice. However, the pathological result of the occupying lesion biopsy specimen in the right lung revealed fibrous tissue hyperplasia with a small amount of inflammatory cell infiltration, while the pathological results of enlarged lymph nodes biopsy specimens were negative. The Multidisciplinary Team discussed the case, and considered it a malignant tumor. Under the agreement of the patient and his family, the thoracic surgeon performed the right upper pulmonary occupying lesion wedge resection and enlarged lymph node excision biopsy assisted by thoracoscopy under general anesthesia. During the operation, it was found that the right lung occupied a large space and was closely attached to the trachea, with local wrapping around the trachea, making it difficult to completely remove. If a total resection surgery is performed, it is highly likely to damage the trachea and cause serious complications. Therefore, a subtotal resection surgery is performed during the operation.

**Figure 2:**
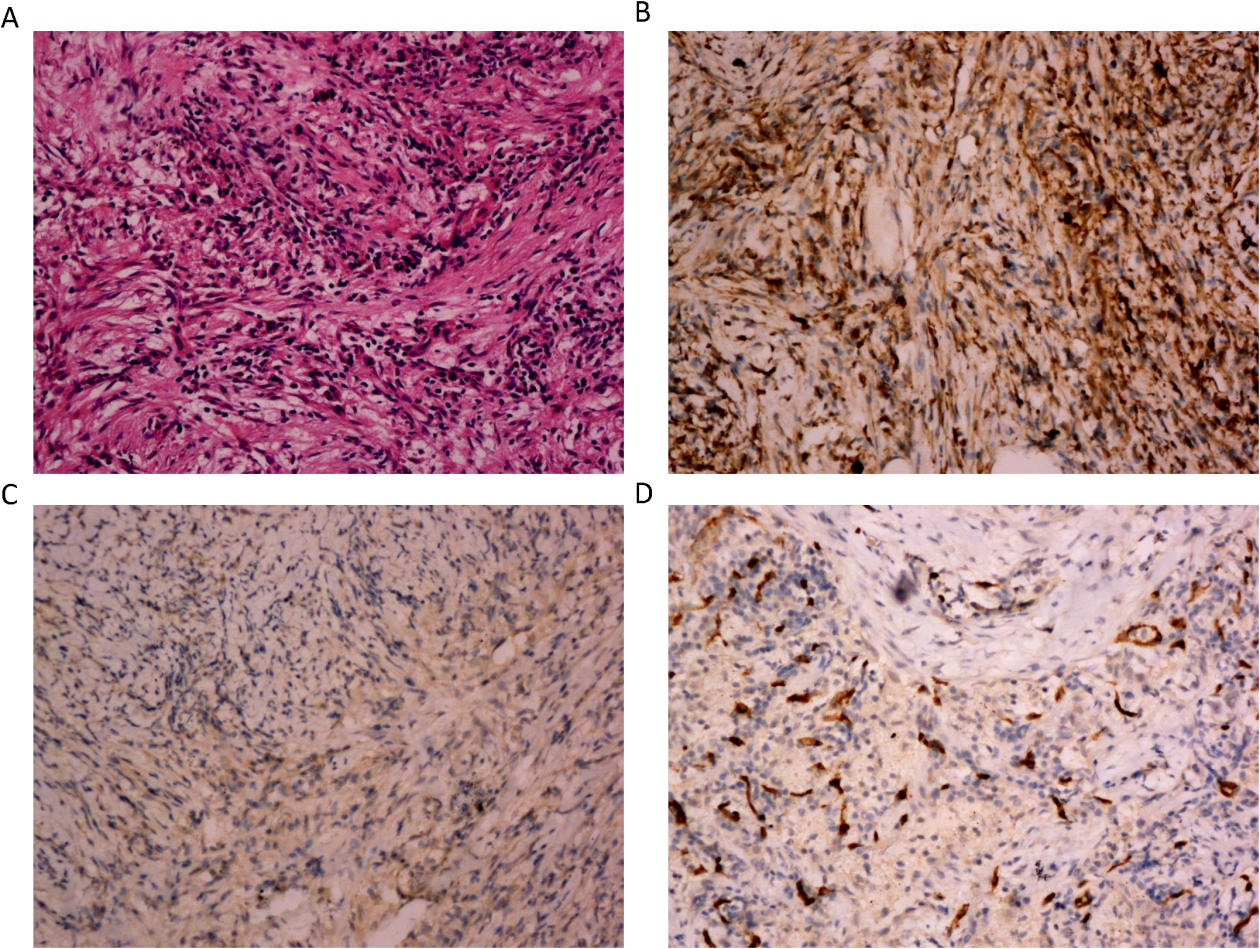
The microphotographs of the histopathological. **(A)** Spindle cells mixed with inflammatory cells. The spindle cells are epithelioid and mixed with chronic inflammatory cells. There is increased vascularity in the IMT. (H&E, 200x). **(B)** Immunostaining positive for vimentin (200x). **(C)** Immunostaining negative for ALK (200x). **(D)** Immunostaining positive for CD34 (200x).

Pathologic diagnosis for this patient revealed that the morphology of “right upper lung mass” was considered as inflammatory myofibroblastic tumor with endovascular changes. Immunohistochemical staining results were as follows: CD163 (+), CD68 (+), ALK (-), Desmin (-), S100 (-), SMA (+) and CD34 (blood vessels +), Vimentin (+) and STAT6 (-), CD23 (-), CD138 (plasma cells +), CD38 (plasma cells +), IgG4 (-), IgG (-), CK (Pan) (alveolar epithelium +), PAS (-), PASM (-), and acid-fast staining (-) (**Figure 2**).

**Figure 3:**
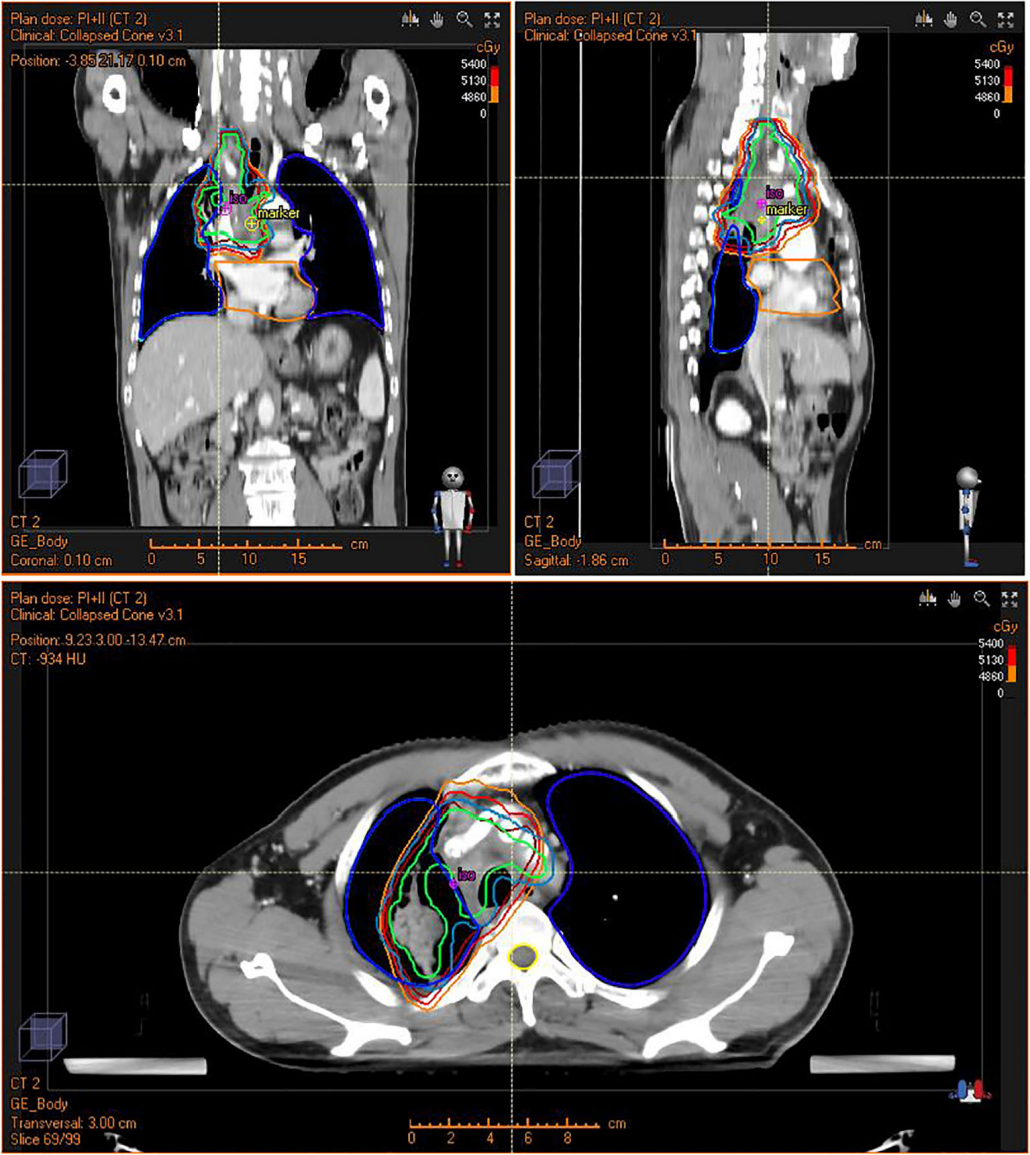
Intensity Modulated Radiation Therapy (IMRT) plan for the patient showing conformal radiation dose delivery to the tumor.

After recovering from the operation, the patient was transferred to the oncology department for antitumor treatment one month after surgery. Because of subtotal resection, the patient accepted concurrent radiochemotherapy. In the first stage, the radiotherapy dose was PTV: DT 3800cGy/19F. Boost CTV: DT 1600cGy/8F in the second stage. The total dose on the tumor was 5400cGy/27F (**Figure 3**). The patient received weekly concurrent chemotherapy with cisplatin dosed at 40mg qw for 5 cycles during radiotherapy. The side effects of concurrent radiochemotherapy were grade I myelosuppression with WBC 3.22 x10^9/L during concurrent radiochemotherapy and grade I radiation pneumonitis 6 months after radiochemotherapy.

After therapy, the IMT did not recur. The patient was given a CT/MRI and hematological index every 3 months and experienced no more adverse events. Progression-free survival (PFS) lasted more than 5 years. Written informed consent was obtained from the patient to publish the case report and related images.

## Discussion and conclusion

We reported an IMT located in the right lung and mediastinum, which was treated with X-beam irradiation and cisplatin after subtotal resection.

IMT is a rare tumor and primarily occurs in soft tissues. Usually, IMT presents as a nodular circumscribed mass, occupying lesion, multinodular lesions in the soft tissue of chest, abdominopelvic or retroperitoneal region (2). The definitive diagnosis of IMT is mainly based on the histopathological report. In this case, the presence of CD163, CD68 SMA, CD34, and Vimentin and the absence of ALK, S-100, Desmin, CD23, STAT6, PAS, PASM, and acid-fast staining associated with the mix of spindle cells and inflammatory cells helped the histopathological diagnosis.

Because IMT always presents local invasiveness, thus local therapy plays an important role, especially surgical resection (6). Besides, complete surgical resection is the standard treatment for localized IMT. However, local recurrences are common with incomplete resection, which usually occurs in the presence of involved surgical margins (10). Typically, about 23-25% of IMT recurred after surgery (11, 12). Nonetheless, a second surgery, radiotherapy, or chemotherapy regimen can be effective in recurrent IMT (13, 14). It is a challenge to prevent recurrence of IMT after surgery.

Because of the inflammatory features of IMT, anti-inflammatory drugs, such as steroids and non-steroidal anti-inflammatory drugs (NSAIDs), were the first applied in the treatment. However, different cases reported opposite results of anti-inflammatory drugs (15, 16). Therefore, the effect of anti-inflammatory drugs in IMT might depend on individual patient differences and tumor heterogeneity.

Chemotherapy is reportedly effective in IMT. Moreover, it is used in neoadjuvant therapy and postoperative adjuvant therapy for unresectable, progressive, or metastatic disease. Multiple chemotherapy regimens have been described as functional, including ifosfamide, carboplatin, vincristine and dactinomycin (IVA), paclitaxel, vincristine, methotrexate and vinblastine (MTX/VBL), and vinorelbine (MTX/VNB) (2, 3). The development of targeted drugs shows a good therapeutic effect in the IMT treatment directed toward ALK, PDGFR β, ROS1, NTRK, RET, and other molecular targets (2, 4). Thus, molecular landscape targeted therapeutics might play a more critical role in IMT. In addition to the recurrent ALK arrangement, ALK negative IMTs are reported with other gene rearrangements such as ROS1, NTRK and RET (17, 18). ROS1 rearrangements have been identified about 10% of IMT. As little is known about the potential oncogenic drivers of ALK-negative tumors, there are still no standard targeted therapies available. In an ALK negative IMT patient with ROS1 gene rearrangement, crizotinib was showed the curative effect (19). Furthermore, NTRK fusion case also showed a response to crizotinib (20). Immunotherapy and tyrosine kinase inhibitors (TRKi) also reported to be an important therapeutic option in NTRK-altered tumors (21, 22). Therefore, we should search for gene mutations as well and consider target treatment options in advanced and recurrent IMTs.

Radiotherapy also showed effectiveness in IMT (4, 23–25). It may be a choice for localized IMT patients who cannot tolerate surgery or could be used as postoperative adjuvant therapy in unresectable, progressive, or metastatic cases. However, there is no standardized radiation dose and combined drug therapy regimen. We summarize the reported radiation used in IMT in **Table 1**.

**Table 1:** Summary of inflammatory myofibroblastic tumor treated with radiotherapy.

Reference	N	Age (y)	Sex	Tumor site	Treatments	Radiotherapy dose	Follow up
Lisi R 2019 (18)	1	26	F	Trachea	FRT	4500cGy/25F	PFS≧62 months
Strianese 2018 (20)	20	5-76	12M+13F	orbit	STL/TR+steroid (22/25) +radiotherapy (3/25)	2000cGy/1F	radiotherapy (1/3) recurred after 13 years
Chennouf 2017 (6)	1	26	M	Left parieto-occipital	Four surgical resections and FRT+crizotinib +ceritinib	6000cGy/ 30F	PFS≧14 months
Gorolay2016 (21)	1	49	M	Posterior mediastinum	thoracic surgical biopsy+external beam radiotherapy	NR	PFS≧36 months
Zhang 2015 (14)	1	49	M	Right inguinal region	Surgery Second Surgery +FRT	4600cGy/23F	PFS≧6 months
Gabel 2015 (19)	1	56	M	Left Sphenoid and Cavernous Sinus	STL +steroid therapy +FRT	2000cGy/10F	PFS≧24 months
Maire 2013 (23)	1	38	M	Skull base	Corticosteroids+FRT	2000cGy/10F	PFS≧24 months
Lee 2006 (22)	8	52-76	4M+4F	Skull base	Steroid therapy (7/8) +low-dose radiation therapy (6/8)	2000cGy (6/8) 3000cGy (1/8)	PFS NR recurrence (7/8) recurrence in low-dose group (5/6)
Present case 2022	1	54	M	right lung, and mediastinum	STL+FRT+Chenmotherapy	5400cGy/27F	PFS≧47 months

Fractionated radiotherapy (FRT), F=female, M=male, NR=not reported STL=subtotal resection, TR=total resection.

It was reported that Low-dose radiation therapy (2000cGy) combined with steroid therapy treatment for IMT in the skull base showed a high recurrence rate (26). Nevertheless, low-dose radiation therapy (2000cGy) treatment for IMT in orbit showed good effect (23). It seems that the radiation dose might vary based on the tumor’s location. Reference the radiotherapy dose for common tumors in the same position might be suitable. However, it remains unknown whether radiotherapy should combine chemotherapy or steroid treatment. The last is usually offered as a combined therapy (4, 24, 27). Chemotherapy, especially cisplatin, might sensitize the patient for the radiation and improve the radiotherapy effect (28). For high-risk patients, chemotherapy combined with radiotherapy might be an intensive treatment.

In our case, radiotherapy and chemotherapy played an essential role in treating this patient with subtotal resection. Despite the local progress and subtotal resection, the progression-free survival (PFS) was more than 5 years. We first reported high-dose radiotherapy combined with chemotherapy treatment for unresectable IMT. In conclusion, concurrent radiochemotherapy may be considered for local progress, local recurrence, and nonresectable IMT patients with high risk.

## Data Availability

The raw data supporting the conclusions of this article will be made available by the authors, without undue reservation.

## References

[1] Jo VY Fletcher CD. WHO classification of soft tissue tumours: an update based on the 2013 (4th) edition. Pathology. (2014) 46:95–104. doi: 10.1097/PAT.0000000000000050 24378391

[2] Mahajan P Casanova M Ferrari A Fordham A Trahair T Venkatramani R. Inflammatory myofibroblastic tumor: molecular landscape, targeted therapeutics, and remaining challenges. Curr Probl Cancer. (2021) 45:100768. doi: 10.1016/j.currproblcancer.2021.100768 34244015

[3] Coffin CM Hornick JL Fletcher CD. Inflammatory myofibroblastic tumor: comparison of clinicopathologic, histologic, and immunohistochemical features including ALK expression in atypical and aggressive cases. Am J Surg Pathol. (2007) 31:509–20. doi: 10.1097/01.pas.0000213393.57322.c7 17414097

[4] Strianese D Tranfa F Finelli M Iuliano A Staibano S Mariniello G. Inflammatory myofibroblastic tumor of the orbit: A clinico-pathological study of 25 cases. Saudi J Ophthalmol. (2018) 32:33–9. doi: 10.1016/j.sjopt.2018.04.001 PMC5943919 29755269

[5] Chennouf A Arslanian E Roberge D Berthelet F Bojanowski M Bahary JP. Efficiency of crizotinib on an ALK-positive inflammatory myofibroblastic tumor of the central nervous system: A case report. Cureus. (2017) 9:e1068. doi: 10.7759/cureus.1068 28409069 PMC5375952

[6] Casanova M Brennan B Alaggio R Kelsey A Orbach D van Noesel MM. Inflammatory myofibroblastic tumor: The experience of the European pediatric Soft Tissue Sarcoma Study Group (EpSSG). Eur J Cancer. (2020) 127:123–9. doi: 10.1016/j.ejca.2019.12.021 32007712

[7] Biswas R Halder A Gangopadhyay M Biswas D. Inflammatory myofibroblastic tumor of maxillary sinus successfully treated with radiotherapy and corticosteroid: report of a rare case. J Egypt Natl Canc Inst. (2020) 32:26. doi: 10.1186/s43046-020-00038-0 32488371 PMC13317098

[8] Inadomi K Kumagai H Takayoshi K Ariyama H Kusaba H Nishie A. Successful combination chemotherapy for metastatic inflammatory myofibroblastic tumor: A case report. Oncol Lett. (2015) 10:2981–5. doi: 10.3892/ol.2015.3708 PMC4665821 26722275

[9] Coffin CM Patel A Perkins S Elenitoba-Johnson KS Perlman E Griffin CA. ALK1 and p80 expression and chromosomal rearrangements involving 2p23 in inflammatory myofibroblastic tumor. Mod Pathol. (2001) 14:569–76. doi: 10.1038/modpathol.3880352 11406658

[10] Mehta B Mascarenhas L Zhou S Wang L Venkatramani R. Inflammatory myofibroblastic tumors in childhood. Pediatr Hematol Oncol. (2013) 30:640–5. doi: 10.3109/08880018.2013.816810 23988029

[11] Alaggio R Cecchetto G Bisogno G Gambini C Calabrò ML Inserra A. Inflammatory myofibroblastic tumors in childhood: a report from the Italian Cooperative Group studies. Cancer. (2010) 116:216–26. doi: 10.1002/cncr.v116:1 19852031

[12] Hussong JW Brown M Perkins SL Dehner LP Coffin CM. Comparison of DNA ploidy, histologic, and immunohistochemical findings with clinical outcome in inflammatory myofibroblastic tumors. Mod Pathol. (1999) 12:279–86. 10102613

[13] Zhang T Yuan Y Ren C Du S Chen J Sun Q. Recurrent inflammatory myofibroblastic tumor of the inguinal region: A case report and review of the literature. Oncol Lett. (2015) 10:675–80. doi: 10.3892/ol.2015.3297 PMC4509014 26622552

[14] Li J Yin WH Takeuchi K Guan H Huang YH Chan JK. Inflammatory myofibroblastic tumor with RANBP2 and ALK gene rearrangement: a report of two cases and literature review. Diagn Pathol. (2013) 8:147. doi: 10.1186/1746-1596-8-147 24034896 PMC3850018

[15] Grünholz D Appiani F Abarca C Manríquez M Pinilla J Wainstein E. Peritoneal myofibroblastic tumor successfully treated with infliximab: Report of one case. Rev Med Chil. (2015) 143:943–7. doi: 10.4067/S0034-98872015000700017 26361033

[16] Su W Ko A O'Connell T Connell T Applebaum H. Treatment of pseudotumors with nonsteroidal antiinflammatory drugs. J Pediatr Surg. (2000) 35:1635–7. doi: 10.1053/jpsu.2000.18340 11083441

[17] Lopez-Nunez O John I Panasiti RN Ranganathan S Santoro L Grélaud D. Infantile inflammatory myofibroblas tic tumors: clinicopathological and molecular characterization of 12 cases. Mod Pathol. (2020) 33:576–90. doi: 10.1038/s41379-019-0406-6 31690781

[18] Lovly CM Gupta A Lipson D Otto G Brennan T Chung CT. Inflammatory myofibroblastic tumors harbor multiple potentially actionable kinase fusions. Cancer Discovery. (2014) 4:889–95. doi: 10.1158/2159-8290.CD-14-0377 PMC4125481 24875859

[19] Mai S Xiong G Diao D Wang W Zhou Y Cai R. Case report: crizotinib is effective in a patient with ROS1-rearranged pulmonary inflammatory myofibroblastic tumor. Lung Cancer. (2019) 128:101–4. doi: 10.1016/j.lungcan.2018.12.016 30642440

[20] Schöffski P Wozniak A Stacchiotti S Rutkowski P Blay JY Lindner LH. Abstract 3191: Detection of molecular drivers in inflammatory myofibroblastic tumor: Study on archival tissue from EORTC 90101 CREATE phase II clinical trial. Cancer Res. (2020) 80:3191. doi: 10.1158/1538-7445.AM2020-3191

[21] Dufresne A Pissaloux D Ngo C Penel N Le Cesne A Macagno N. Natural history and treatment efficacy in an ambispective case series of NTRK-rearranged mesenchymal tumors. ESMO Open. (2023) 8:101202. doi: 10.1016/j.esmoop.2023.101202 37054503 PMC10163158

[22] Demetri GD Antonescu CR Bjerkehagen B Bovée JVMG Boye K Chacón M. Diagnosis and management of tropomyosin receptor kinase (TRK) fusion sarcomas: Expert recommendations from the World Sarcoma Network. Ann Oncol. (2020) 31:1506–17. doi: 10.1016/j.annonc.2020.08.2232 PMC7985805 32891793

[23] Lisi R Abate G D'Urso P Martinetti MT Siniscalchi B Marampon F. Successful role of adjuvant radiotherapy in a rare case of tracheal inflammatory myofibroblastic tumor: a case report. Tumori. (2019) 105:NP1–1NP3. doi: 10.1177/0300891619838333 30900517

[24] Gabel BC Goolsby M Hansen L U HS. Inflammatory myofibroblastic tumor of the left sphenoid and cavernous sinus successfully treated with partial resection and high dose radiotherapy: case report and review of the literature. Cureus. (2015) 7:e328. doi: 10.7759/cureus.328 26543686 PMC4627831

[25] Gorolay V Jones B. Inflammatory myofibroblastic tumor of mediastinum with esophageal and bronchial invasion: a case report and literature review. Clin Imaging. (2017) 43:32–5. doi: 10.1016/j.clinimag.2016.09.012 28178581

[26] Lee DK Cho YS Hong SH Chung WH Ahn YC. Inflammatory pseudotumor involving the skull base: response to steroid and radiation therapy. Otolaryngol Head Neck Surg. (2006) 135:144–8. doi: 10.1016/j.otohns.2006.01.016 16815200

[27] Maire JP Eimer S San Galli F Franco-Vidal V Galland-Girodet S Huchet A. Inflammatory myofibroblastic tumour of the skull base. Case Rep Otolaryngol. (2013) 2013:103646. doi: 10.1155/2013/103646 23573442 PMC3614032

[28] Hennequin C Guillerm S Quero L. Combination of chemotherapy and radiotherapy: A thirty years evolution. Cancer Radiother. (2019) 23:662–5. doi: 10.1016/j.canrad.2019.07.157 31473087

